# Structure of a *Burkholderia pseudomallei* Trimeric Autotransporter Adhesin Head

**DOI:** 10.1371/journal.pone.0012803

**Published:** 2010-09-20

**Authors:** Thomas E. Edwards, Isabelle Phan, Jan Abendroth, Shellie H. Dieterich, Amir Masoudi, Wenjin Guo, Stephen N. Hewitt, Angela Kelley, David Leibly, Mitch J. Brittnacher, Bart L. Staker, Samuel I. Miller, Wesley C. Van Voorhis, Peter J. Myler, Lance J. Stewart

**Affiliations:** 1 Seattle Structural Genomics Center for Infectious Disease (SSGCID), Seattle, Washington, United States of America; 2 Emerald BioStructures, Bainbridge Island, Washington, United States of America; 3 Seattle Biomedical Research Institute, Seattle, Washington, United States of America; 4 School of Medicine, University of Washington, Seattle, Washington, United States of America; 5 Departments of Microbiology, Medicine, Genome Sciences, and Immunology, University of Washington, Seattle, Washington, United States of America; University of Oulu, Germany

## Abstract

**Background:**

Pathogenic bacteria adhere to the host cell surface using a family of outer membrane proteins called Trimeric Autotransporter Adhesins (TAAs). Although TAAs are highly divergent in sequence and domain structure, they are all conceptually comprised of a C-terminal membrane anchoring domain and an N-terminal passenger domain. Passenger domains consist of a secretion sequence, a head region that facilitates binding to the host cell surface, and a stalk region.

**Methodology/Principal Findings:**

Pathogenic species of *Burkholderia* contain an overabundance of TAAs, some of which have been shown to elicit an immune response in the host. To understand the structural basis for host cell adhesion, we solved a 1.35 Å resolution crystal structure of a BpaA TAA head domain from *Burkholderia pseudomallei*, the pathogen that causes melioidosis. The structure reveals a novel fold of an intricately intertwined trimer. The BpaA head is composed of structural elements that have been observed in other TAA head structures as well as several elements of previously unknown structure predicted from low sequence homology between TAAs. These elements are typically up to 40 amino acids long and are not domains, but rather modular structural elements that may be duplicated or omitted through evolution, creating molecular diversity among TAAs.

**Conclusions/Significance:**

The modular nature of BpaA, as demonstrated by its head domain crystal structure, and of TAAs in general provides insights into evolution of pathogen-host adhesion and may provide an avenue for diagnostics.

## Introduction

*Burkholderia pseudomallei* and *Burkholderia mallei* are closely related gram-negative bacteria that are the causative agents of melioidosis and glanders, respectively. These organisms are considered biothreat agents and are classified by the NIAID as class B pathogens. Using bacteriophage-mediated immunoscreening, Tiyawisutsri *et al.* identified four Trimeric Autotransporter Adhesins (TAAs) in *B. mallei* that were expressed during glanders infection[1]. TAAs are a family of outer membrane proteins that adhere to host cell surfaces, and thus have an important role in virulence of these pathogens [2]. Because TAAs are surface proteins with properties similar to hemagglutinins and invasins, these proteins are also referred to as Hep_Hag autotransporters or YadA-like autotransporters, in reference to the first known member of the family. Because of the differences in protein folds and binding partners between hemagglutinins and this family of proteins, we have adopted the more common and less confusing name of TAA [3]. All four TAAs identified by Tiyawisutsri *et al.* have homologs in *B. pseudomallei*. In total six TAAs were identified in *B. mallei* and nine in *B. pseudomallei*, indicating that these proteins are in relative abundance in comparison with other bacteria. TAAs have also been identified in *Burkholderia cenocepacia*, a respiratory pathogen associated with cystic fibrosis [4].

The earliest known and most well characterized TAA is YadA from *Yersinia enterocolitica* [5]. YadA is comprised of a C-terminal membrane anchored domain and an N-terminal domain, which is referred to as a passenger domain because it is believed to pass through the membrane anchored domain on its way outside the cell; hence, the term autotransporter. Based on the YadA primary structure, passenger domains are comprised of an N-terminal secretion sequence, a head region, and a stalk region. The head of YadA has been shown to bind collagen [6]. A crystal structure of the head domain of YadA contains a proposed collagen binding surface [7], and YadA appears to bind to the triple-helical structure of collagen without sequence specificity [8]. Although a structure of an entire TAA has not yet been solved, structures have also been determined for individual domains of other TAAs. These include several parts of the head domains of Hia from *Haemophilus influenza* [9], [10] and BadA from *Bartonella henselae* [11]; the coiled-coil domains from YadA [12], *Salmonella enterica* [13], and UspA1 from *Moraxella catarrhalis* [14]; and the membrane anchoring domain of Hia [9], [15]. A recent crystal structure of the entire esterase EstA non-trimeric autotransporter from *Pseudomonas aeruginosa* provides additional insights into autotransporter function [16].

In general, TAAs are highly variable in sequence and length, making them difficult to identify and define their domain boundaries [3]. The most highly conserved and identifiable region of a TAA is the membrane anchored C-terminal region, often referred to as the YadA domain. Outside of this domain, it is challenging to identify other whole domains using general domain approaches. In contrast, approaches that identify short sequence motifs or subdomains of up to 40 amino acids in length are considerably more successful [3]. These short sequence motifs include the N-terminal secretion sequence, neck regions which may contain up to 50% sequence identity [11], left handed parallel β-roll repeats (also referred to as Hep_Hag, YadA-like head or Ylhead repeats), and other elements not present in general domain databases. However, the regions surrounding these sequence motifs often contain low sequence similarity complicating the prediction of these elements and their boundaries. In one example, crystal structures of the Hia and BadA head domains reveal nearly identical folds for the Trp-ring and GIN domains despite no discernable sequence similarity *a prior* [11]. In another example, Hia contains three Trp-ring elements which as little as 18% sequence identity, but virtually super-imposable folds [9]. Despite low sequence similarity, these structures indicate the rules for the structural assembly of these domains. Several other short sequence motifs appear throughout TAAs that do not yet have known three dimensional structures. Structure elucidation of these motifs should aid in the development of new bioinformatic algorithms to identify these motifs in other TAAs.

In comparison with YadA, the nine TAAs from *B. pseudomallei* are considerably larger and more complex. Several *B. pseudomallei* TAAs contain multiple head regions, regions of low complexity, and are up to 2800 amino acids long [1]. To understand how *B. pseudomallei* binds to its host cell surface, we have investigated several *B. pseudomallei* TAAs using a structural genomics approach [17], [18], [19], [20]. Here we present the crystal structure of a head domain from a *B. pseudomallei* trimeric autotransporter adhesin BpaA at 1.35 Å resolution. The BpaA head structure exhibits a novel fold of an intricately interwoven trimer that contains modular structural elements from other trimeric autotransporter proteins. Our work expands the foundation for understanding the structural basis for the adherence of infectious disease organisms to their hosts via TAAs.

## Results

### BpaA Domain Architecture

**Figure 1:**
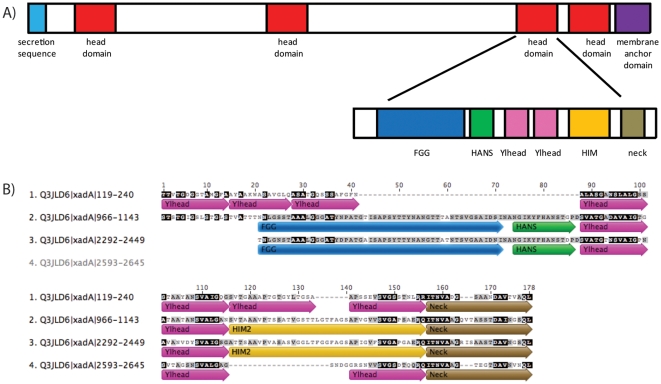
Primary structure and domain annotation of BpaA from *Burkholderia pseudomallei*. A. Domain architecture of the *B. pseudomallei* BpaA trimeric autotransporter adhesin (TAA). The BpaA TAA features an N-terminal secretion sequence, four head domains, and a C-terminal membrane anchored domain. The residues between head domains are identified as regions of low complexity and the region between the fourth head domain and the membrane anchored domain is likely to be a coiled-coil. The third head domain contains numerous sequence motifs identified by the domain annotation of Trimeric Autotransporter Adhesins (daTAA) server [3] B. Multiple sequence alignment of four head domains of *B. pseudomallei* BpaA The four head domains were aligned according to their sequence and motifs identified by the daTAA are indicated.

The genome of *B. pseudomallei* contains a gene annotated as xadA or XadA-like protein based on sequence similarity to the TAA XadA from *Xanthomonas oryzae* [21]. To avoid confusion with the XadA protein from *X. oryzae*, we have adopted the term BpaA for the *Burkholderia pseudomallei* adhesion A protein investigated here. In *B. pseudomallei*, BpaA TAA is expressed as a 2757 amino acid long protein containing an N-terminal secretion peptide sequence, numerous head domains, a short coiled-coil domain, and a C-terminal membrane anchor domain (Figure 1). Pfam [22] predicted the third head domain of BpaA to contain Hep_Hag and HIN2 domains (BPSS1434) [1]. To identify domain boundaries in the *B. pseudomallei* TAAs, the sequences were analyzed with InterPro [23] and the resulting Hep_Hag and HIN2 domains were aligned to the folded region of the YadA crystal structure [7] using the MUSCLE algorithm [24] iteratively, with re-alignment after each manual sequence extension or truncation. Ginzu [25] domain boundary predictions suggested that the full-length protein was all beta, even within the low-complexity segments. DISOPRED did not predict any disordered regions [26]. In addition, the entire BpaA sequence was entered into the domain annotation of trimeric autotransporter adhesins (daTAA) server [3] which predicted this region of BpaA to contain an FGG motif, a HANS motif, two Ylhead repeats, a HIN2 motif, a neck motif, and end with a coiled-coil (Figure 1). With this combined information set, domain boundaries were manually selected to contain the aforementioned region resulting in a 178 amino acid long construct that spanned residues 2278–2455. Alignment of this sequence with the other three *B. pseudomallei* BpaA head regions (159–240, 39% sequence identity; 972–1148, 73%; and 2482–2653, 29%) matched reasonably well with the domain boundaries predicted by the daTAA server despite differences in the presence and number of TAA sequence motifs (119–240, 966–1143, and 2583–2645; Figure 1). Of those sequence motifs contained within this head region of BpaA, only the Ylhead and neck motifs have known structures from TAA homologs.

### Crystallization and Structure Determination

Crystallization trials were attempted using a rational sparse matrix approach [27] with a construct containing residues 2278–2455 including a 21 amino acid long N-terminal affinity tag (see methods). Crystals were not observed for this construct, and therefore we tried *in situ* proteolysis using chymotrypsin to generate crystals [28]. Chymotrypsin cleaves after hydrophobic residues, which in this case is most likely after leucine 4 of the target protein sequence. Cleavage at this site would eliminate the 21 amino acid long affinity tag plus the first four amino acids of the target protein sequence. Although we do not know if *in situ* proteolysis was successful, crystals were not obtained in the absence of chymotrypsin. The crystals belonged to the rhombohedral space group *H*3 with unit cell dimensions (Table 1) consistent with one molecule in the asymmetric unit as predicted by the packing density [29], [30]. A 1.35 Å resolution native data set was collected at the Advanced Light Source synchrotron (Table 1).

**Table 1:** Crystallographic data and refinement statistics.

Parameters	Native data set	Iodide data set
**Crystallographic statistics**		
**cell parameters**	a = b = 50.72 Å	a = b = 50.78 Å
	c = 136.50 Å	c = 135.66 Å
	α = β = 90, γ = 120°	α = β = 90, γ = 120°
**Space group**	*H*3	*H*3
**Wavelength used**	0.9765 Å	1.5418 Å
**Resolution** a	50–1.35 Å (1.39−1.35 Å)	20–2.05 Å (2.09−2.05 Å)
**Number all reflections**	303,575	88,646 (4329)
**Number unique reflections**	28,462 (1196)	16,334 (1196)
**Completeness**	98.9% (81.7%)	99.5% (96.0%)
**<I>/<σ(I)>**	19.5 (2.21)	53.75 (30.97)
**R_merge_**	0.075 (0.413)	0.025 (0.039)
**Multiplicity**	4.8 (2.8)	5.4 (3.6)
**Mosaicity**	0.4	0.25
**Figure of Merit**		0.49 (0.42)
**Refinement statistics**		
**R_work_**	11.5%	11.7%
**R_free_** b	12.8%	15.4%
**r.m.s.d. bond length**	0.007	0.010
**r.m.s.d. bond angles**	1.116	1.142
**Average B-factor**	16.3 Å^2^	7.8 Å^2^
**Ramachandran Plot**		
**Residue in favored region**	170 (98.8%)	162 (98.2%)
**Residues in allowed region**	172 (100%)	165 (100%)
**Residues in disallowed region**	0 (0%)	0 (0%)
**Molprobity Score**	0.66 (100^th^ percentile)^c^	1.11 (100^th^ percentile)
**PDB ID**	3LAA	3LA9

a values in parenthesis are for the highest resolution shell.

b 5% of the reflections were omitted and used for calculation of R_free_.

c 100^th^ percentile is best among structures of comparable resolution (±0.25 Å) at time of deposition [33].

Attempts at molecular replacement using elements from YadA, Hia and Dex49a that have weak sequence similarity to BpaA as search models were unsuccessful. Therefore, we attempted *de novo* phasing. The third head region of BpaA does not contain methionine or cysteine residues, preventing structure determination via single or multiple wavelength anomalous dispersion methods (SAD/MAD) using selenomethionine or covalent heavy metal derivatization via mercury or platinum. Therefore, we attempted iodide ion soaking for SAD experimental phasing [31], [32], which has proven successful for several other SSGCID targets (PDB entries 3K9G, 3KM3, 3KW3, 3LR0, 3LR5, 3LUV, 3MEN, 3MD7). A 2.05 Å resolution data set was obtained in house on a crystal soaked into a solution containing 1M potassium iodide (Table 1). Noting the twin fraction of 0.2, the number of iodide sites selected for phase determination was kept to a minimum to avoid including strong sites from the minor twin fraction. Four iodide sites were located for phase determination (see Methods). Each of these sites contained strong anomalous signal. Using the iodide/SAD experimental phases, the model was built initially using automated programs followed by manual model building. Of the 178 amino acid long construct, residues Ser5-Ala122 and Phe129-Asn177 were modeled. Although only 4 sites were used in determination of the experimental phases, a total of 9 iodide sites were located and built based on anomalous Fourier maps. All of the iodide ions bind to the surface of BpaA primarily at hydrophobic pockets, except for one iodide ion which binds along a 3-fold crystallographic axis within the coiled-coil at the C-terminus. The coiled-coil regions of other TAAs have been reported to bind ions [13]. The isomorphous native data set was refined directly against the protein only model from the iodide-derived structure. Two additional residues could be built in the native data set including Ser123 and Ser178. Both structures are well refined with excellent geometry (Table 1) as determined by MolProbity [33].

### BpaA head domain structure

**Figure 2:**
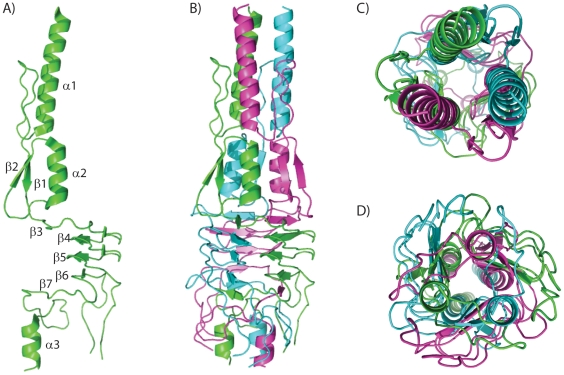
Overall structure of *B. pseudomallei* BpaA third head domain. A. Monomeric structure of the third head domain of BpaA B. Quaternary trimeric structure of third head domain of BpaA C. Trimer top down view D. Bottom-up view.

The structure of the head domain of BpaA exhibits a tightly woven trimeric quaternary structure (Figure 2). No structural homolog could be found using the secondary structure matching (SSM) server [34] and thus, BpaA has a novel fold. The fold has less α/β character than most soluble proteins with numerous loop regions of undefined secondary structure. α1 stacks below α2′ of an adjacent monomer, while α2 stacks on top of α1′′ from the third monomer of the trimeric complex (Figure 2B and 2C). This combined α-helical stack is offset by 60° relative to α3 at the C-terminus (Figure 2C and 2D). β1 stacks against α2 and α2′ on the interior and β2 on the exterior. β3 follows α2 and forms the first β sheet in a stack with β4′, β5′, β6′, and β7 on the interior of a left handed parallel β-roll [7]. The three copies of α1 (i.e. α1, α1′, and α1′′) form a left handed heptad coiled-coil at the N-terminus of this third head domain of BpaA. However the sequence does not strictly follow the standard hxxhcxc pattern where h is a hydrophobic residue, c is a charged residue and x is another amino acid [35] since there are no charged residues in this stretch from Ile8 through Ser21.

**Figure 3:**
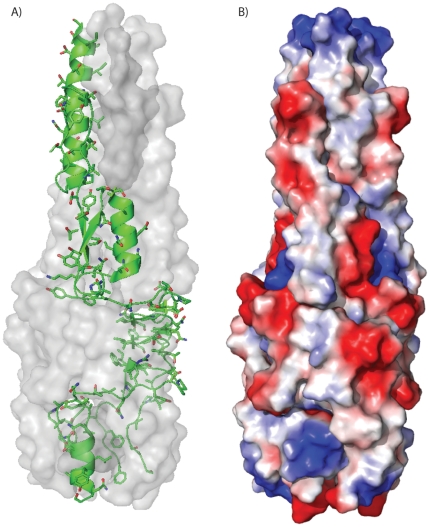
Surface and interior structure of *B. pseudomallei* BpaA third head domain. A. One monomer of the BpaA head is sown in green ribbons with side chains shown in stick representation, while the other two monomers of the trimer are shown as a translucent molecular surface rendering in gray. B. Electrostatic surface potential mapped onto a molecular surface rendering of the third BpaA head domain. Blue indicates regions of positive charge and red indicates regions of negative charge.

Like other TAA head regions [11], the monomer is unlikely to be folded in the absence of the other two protomers of the trimer. Greater than half of the protein is buried by trimer formation given a solvent accessible surface area of 16,298 Å^2^ and a buried surface area of 18,583 Å^2^. The interior of the protein along the 3-fold trimer axis is highly hydrophobic (Ile8, Ile11, Thr15, Gly19, Thr22, Leu26, Tyr44, Val59, Ile63, Ile66, Ile71, Phe74, Ile93, Val105, Ile107, Phe141, Val143, Ile152, Val155, Ala167, Gly170, and Leu173; Figure 3A). The exterior of the protein is hydrophilic (Figure 3A) with a large acidic patch that spans the length of the protein, reflective of the predicted isoelectric point of 4.3 (Figure 3B). The head domain structure of Hia (HiaBD1) also contains an acidic patch [9], [10].

**Figure 4:**
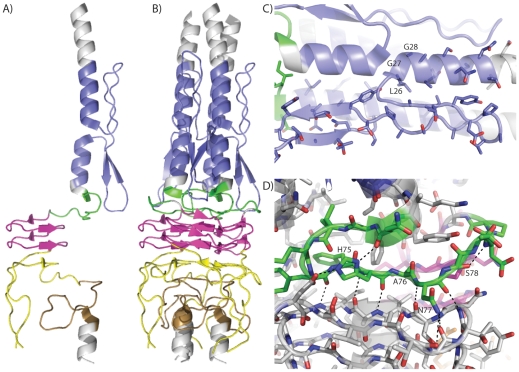
Sequence motifs in the third head domain of *B. pseudomallei* BpaA Trimeric Autotransporter Adhesin (TAA). Coloring indicates sequence motifs identified by the daTAA [3] which is the same as in Figure 1. A) BpaA third head domain monomer B) BpaA third head domain trimer C) FGG motif D) HANS motif. Hydrogen bonds shown as dashed lines.

Bioinformatics approaches [3] predicted several sequence motifs in the *B. pseudomallei* BpaA third head domain (Figure 1) which were mapped onto the BpaA crystal structure (Figure 4). In this case, the sequence motifs predicted by the daTAA server overlap remarkably well with the sequence elements defined by the structure. One of these sequence motifs is the Ylhead (YadA-like head) left handed parallel β-roll, which was observed previously in the YadA head crystal structure [7]. Several sequence motifs or elements predicted from sequence and bioinformatics approaches [3] such as the FGG motif and the HANS motif were novel to the BpaA crystal structure (Figure 4). The structure explains why the few amino acids that define these motifs are highly conserved, whereas other residues within the motif are not well conserved. For example, the FGG motif contains cross-monomer stacked α-helices (α1 with α2′) with a β-turn insert (β1–β2) (Figure 4). FGG motifs typically contain a phenylalanine as the first residue, although BpaA contains the less common leucine residue at this position; twenty-seven of seventy-three sequences aligned in daTAA contain leucine [3]. The first residue (Leu26) must be highly hydrophobic to promote packing at the trimer interface and only appears as phenylalanine or leucine. Modeling a tryptophan or tyrosine residue at this site appears to induce a steric clash along the 3-fold trimeric axis, explaining why these residues are not observed at this position in this sequence motif. The next two residues (27–28) can only be glycine, which would otherwise clash with the helical stacking of α1-α2′ or with the packing of β2 with α2-α2′. These residues are typically followed by the sequence gAxY (details are described on the daTAA server [3]). From this, Tyr32 stacks against α1 while forming a water-mediated interaction with Thr16. The end of α2 includes three additional modestly conserved residues. Ser58 is conserved as a Ser or Asn and induces a turn by forming a hydrogen bond with the backbone of Ala61. Val59 packs within the trimer interface. Gly60 is conserved as a small residue (Gly or Ala).

The HANS motif forms the first β-sheet in the stack of β3, β4′, β5′, β6′, and β7 along the left handed parallel β-roll and forms four backbone hydrogen bonds with β4′ (Figure 4). The side chain of His75 forms hydrogen bonds with Asp82 and the backbone oxygen of Asn69. Ala76 resides in a small pocket created by the highly conserved Tyr73′′ and Phe74′′ that would not accommodate larger amino acids. The side chain of Asn77 forms hydrogen bonds with the backbone oxygen atoms of Thr86 and Gly87. Finally, Ser78 forms a hydrogen bond with the backbone nitrogen of Asp80, forming a turn that leads into the first Ylhead repeat. Thus, with the exception of the His75-Asp82 salt bridge, the HANS motif exclusively forms hydrogen bonding interactions with the backbone atoms of neighboring amino acids. Furthermore, the hydrogen bonding and β-sheet patterns explain why HANS motifs are only found preceding Ylhead repeats [3]. Finally, our structure contains the HIN2 region which has not previously been structurally characterized, although part of this region is disordered (residues 124–128). This region contains a β-sheet which stacks in left-handed parallel β-roll-like fashion on top of the previous Ylhead motif. Inserted within this region is a loop that packs against the neck motif, explaining why the HIN2 motif is only observed prior to the neck motif [3]. This motif is characterized by an FxG motif, two of which is observed back to back from Phe129 through Gly134 in BpaA. Both phenylalanine residues pack against α3, the coiled-coil that extends from the neck motif. We suspect that if our construct had a longer coiled-coil on the C-terminal end of the construct, the turn in the HIN2 domain would have been ordered.

## Discussion

**Figure 5:**
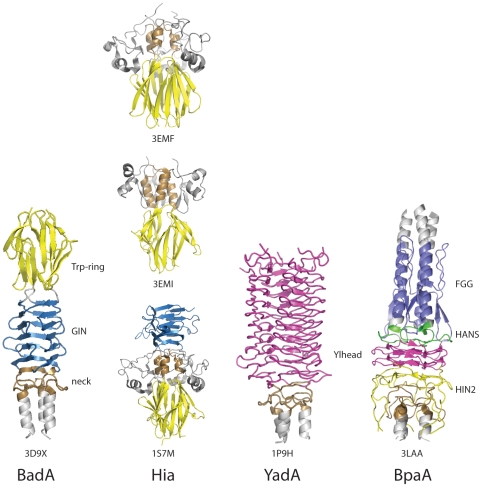
Comparison of architecture of TAA head domain sequence motifs. Trimeric structures are shown for the TAA head domains of BadA from *Bartonella henselae* (PDB ID 3D9X [11]), HiaBD2 (PDB ID 3EMF [9]), KG1-W3 (PDB ID 3EMI [9]), and HiaBD1 (PDB ID 1S7M [10]), from *Haemophilus influenzae*, YadA from *Yersinia enterocolitica* (PDB ID 1P9H [7]), and BpaA from *B. pseudomallei* (PDB ID 3LAA). Trp-ring motifs are shown in yellow, GIN motifs are shown in light blue, neck regions are shown in brown, left handed β-roll Ylhead repeats are shown in magenta, FGG motifs are shown in dark blue, HANS motifs are shown in green, HIN2 motifs are shown in orange, and other regions in gray.

The third head domain of BpaA exhibits a highly interwoven trimeric fold in a manner reminiscent with other known TAA head domain structures YadA [7], Hia [9], [10], and BadA [11]. These different TAAs achieve their tightly interwoven trimeric structures despite considerable sequence divergence and different domain architecture. For each TAA the monomer is composed of several sequence motifs (Figure 5). In the case of BpaA, the Ylhead and neck motifs have been observed in other TAA structures (Figure 5). The collagen binding Ylhead regions, with its characteristic left handed parallel β-roll and SVAIG-S sequence motif, align well between YadA and BpaA. This motif is present in the BadA head domain as well, although it was not part of the construct used for structure determination. In particular, the neck regions align well in structure and contain considerable sequence identity as previously analyzed [9], [11]. The FGG, HANS and HIN2 motifs present in BpaA were not present in YadA, Hia or BadA. Our structure is in agreement with the bioinformatics of these modular elements and explains sequence and domain architecture conservation.

Like other TAAs, a considerable amount of the BpaA head domain does not have α-helix/β-sheet secondary structure. These regions prove difficult to identify by secondary structure prediction programs. As described previously, short sequence motif-based prediction methods fair better at predicting these motifs than general domain prediction methods [3]. The crystal structure of the third head domain from BpaA demonstrates the structural basis of three TAA sequence motifs of previously unknown structure. The structural basis demonstrated for the FGG, HANS and HIN2 sequence motifs by our BpaA structure should be useful for defining sequence restraints within structure prediction programs. In general, TAA sequence motifs are highly modular in nature and often rearranged in different TAAs, yet the structures of sequence motifs such as the Trp-ring motif remain highly similar despite considerable sequence divergence [9], [11]. We predict that the same will hold true for the FGG, HANS, and HIN2 motifs described here once additional structures have been determined.

In comparison with the low sequence identity yet similar structural elements of other TAAs, BpaA may provide additional evidence for evolution of TAAs from a common ancestor [2], [36]. We speculate that this may be common for other extracellular proteins or extracellular domains of membrane-anchored proteins. For example, periplasmic domains of two-component system histidine kinases have evolved to recognize a wide variety of different ligands, yet all exhibit a common PAS domain fold [37], [38]. In addition, lipocalins are extracellular iron binding proteins that exhibit the same fold despite almost no sequence identity in an effort to evade host immune response [39]. Thus, TAAs from pathogenic organisms may exhibit similar general features to other extracellular proteins or extracellular domains of membrane-anchored proteins in terms of evolution and efforts to combat the immune response of their host.

Despite the fact that TAAs perform an important role in host cell infection, it appears that they have evolved with a high degree of sequence divergence and general architecture as a means to escape host immune response. Nevertheless, we note that several domains with highly different sequences exhibit the same fold across several TAAs from pathogenic organisms, perhaps due to a common ancestor. Therefore, antibodies or aptamers that target the global fold rather than the local structure of TAA head domains may hold potential as novel diagnostics.

## Materials and Methods

### Protein expression and purification

The third head domain of BpaA from *B. pseudomallei* strain 1710b (NCBI: YP 335617.1; gene BURPS1710B_A0459; UniProt Q3JLD6) spanning residues 2278 to 2455 was cloned into a pAVA0421 vector using ligation independent cloning (LIC) [40]. BpaA was expressed in *E. coli* using BL21(DE3)R3 Rosetta cells and autoinduction media [41] in a LEX Bioreactor. The frozen cells were resuspended in 200 ml of Lysis Buffer (20 mM HEPES, pH 7.3, 300 mM NaCl, 5% glycerol, 30 mM Imidazole, 0.5% CHAPS, 10 mM MgCl_2_, 3 mM β-mercaptoethanol, 25 units/ml of Benzonase® nuclease, and 0.05 mg/ml lysozyme). The resuspended cell pellet was disrupted on ice for 15 minutes with a Branson Digital Sonifier 450D (settings at 70% amplitude, with alternating cycles of five seconds of pulse-on and ten seconds of pulse-off). The cell debris was clarified by centrifugation on a Sorvall RC5 at 6,000 RPM for 60 min at 4°C. The protein was purified from the clarified cell lysate by immobilized metal affinity chromatography binding on Ni Sepharose High Performance resin (GE Biosciences, Piscataway, NJ) equilibrated with Binding Buffer (20 mM HEPES, pH 7.2–7.4, 300 mM NaCl, 5% glycerol, 30 mM Imidazole). The recombinant protein was eluted with 500 mM imidazole and was further resolved by size-exclusion gel chromatography (SEC, Superdex 75 26/60; GE Biosciences, Piscataway, NJ). Pure fractions collected in SEC Buffer (20 mM HEPES pH 7.0, 300 µM NaCl, 2 mM DTT, and 5% glycerol) as a single peak were pooled. The protein was concentrated, flash frozen, and stored in −80°C. The affinity tag was not removed prior to crystallization trials.

### Crystallization

Crystals were grown using the sitting drop vapor diffusion method at 16 °C using either the JCSG+ or PACT sparse matrix screens from Emerald BioSystems [27]. For the native crystal, 0.4 µL of the protein stock solution at 24.9 mg/mL in 20 mM HEPES, 0.5 M NaCl, 5% Glycerol, 2 mM DTT (pH 7.0) with 0.1 mg/mL chymotrypsin was incubated with a similar volume of reservoir solution (10% PEG 1000 and 10% PEG 8000). The crystal was cryo-protected in the reservoir solution enhanced with 25% ethylene glycol. The crystal used for phase determination was grown against a reservoir of 0.1 M MIB (malonic acid, imidazole, boric acid) buffer pH 4.0 and 25% PEG 1500 and then soaked into a solution containing 0.1 M MIB buffer pH 4.0, 1.0 M KI and 35% PEG 1500 for 1 hour prior to vitrification.

### Data collection

A 1.35 Å resolution native data set was collected remotely at the Advanced Light Source in Berkeley, CA USA using beamline 5.0.3 which has an ADSC Q315 detector. The data were reduced with HKL2000 [42] yielding a rhombohedral space group with one molecule in the asymmetric unit. Crystallographic statistics are presented in Table 1. A 2.05 Å resolution iodide data set was collected in house using a Rigaku Micromax 007-HF X-ray generator with Osmic VariMax HF optics and a Saturn 944 CCD detector. The data were reduced with XDS and XSCALE [43] yielding a data set in the rhombohedral space group isomorphous with the native crystal. The resolution of the iodide data set was limited by the size of the detector and the parameters used for data collection. Both the native and iodide crystals had a twin fraction of about 0.1–0.2 as determined by detwin analysis in the CCP4 suite [44].

### Structure determination

The structure was solved by the single wavelength anomalous dispersion (SAD) method using iodide ions as the heavy atoms. Four iodide sites were located using Phenix [45]. Experimental phasing was performed using PHASER EP from the CCP4 suite [44]. Experimental electron density maps were density modified in PARROT. The initial model was built with BUCCANEER [46] and followed with additional building in ARP/wARP [47]. Additional iodide sites were identified using anomalous difference Fourier maps, and all iodide site occupancies were determined by refinement of the SAD data directly within REFMAC [48]. The final model was produced after numerous reiterative rounds of refinement in REFMAC and manual building in Coot [49]. In later rounds of refinement, the model was refined with amplitude based twin refinement. Refinement statistics are detailed in Table 1.
